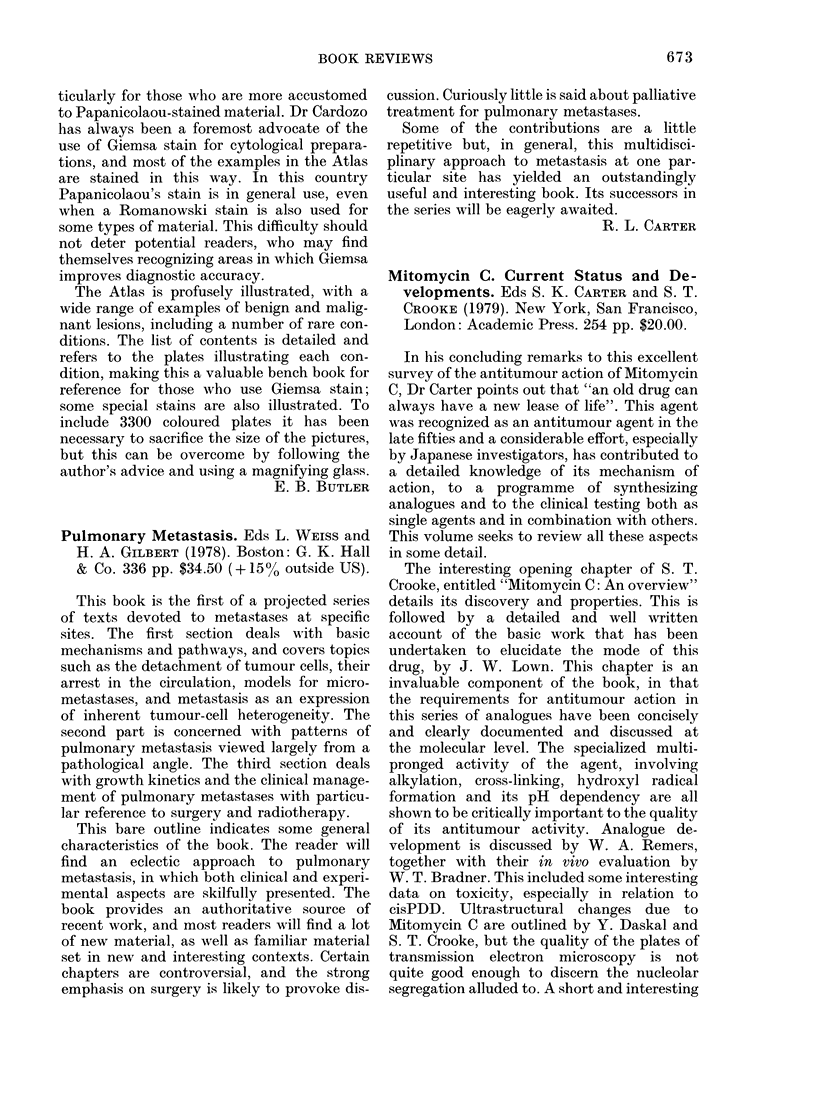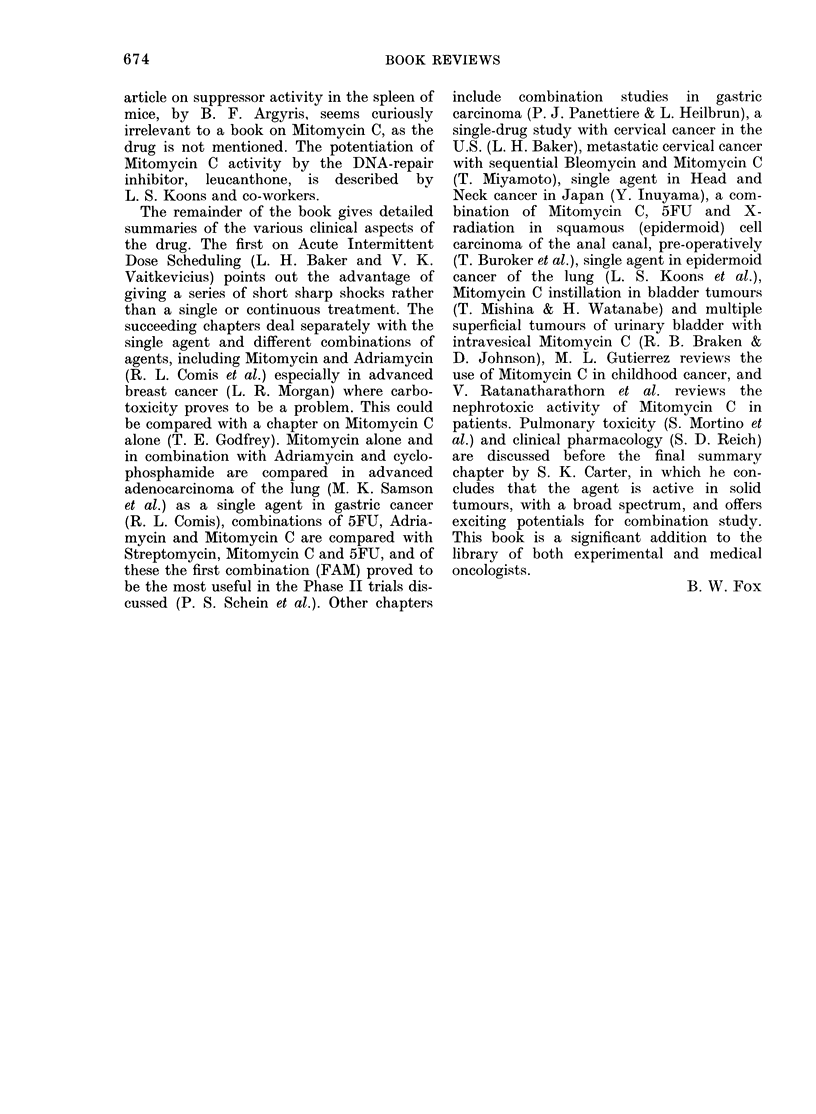# Mitomycin C. Current Status and Developments

**Published:** 1980-04

**Authors:** B. W. Fox


**Mitomycin C. Current Status and Developments.** Eds S. K. Carter and S. T. Crooke (1979). New York, San Francisco, London: Academic Press. 254 pp. $20.00.

In his concluding remarks to this excellent survey of the antitumour action of Mitomycin C, Dr Carter points out that "an old drug can always have a new lease of life". This agent was recognized as an antitumour agent in the late fifties and a considerable effort, especially by Japanese investigators, has contributed to a detailed knowledge of its mechanism of action, to a programme of synthesizing analogues and to the clinical testing both as single agents and in combination with others. This volume seeks to review all these aspects in some detail.

The interesting opening chapter of S. T. Crooke, entitled "Mitomycin C: An overview" details its discovery and properties. This is followed by a detailed and well written account of the basic work that has been undertaken to elucidate the mode of this drug, by J. W. Lown. This chapter is an invaluable component of the book, in that the requirements for antitumour action in this series of analogues have been concisely and clearly documented and discussed at the molecular level. The specialized multipronged activity of the agent, involving alkylation, cross-linking, hydroxyl radical formation and its pH dependency are all shown to be critically important to the quality of its antitumour activity. Analogue development is discussed by W. A. Remers, together with their *in vivo* evaluation by W. T. Bradner. This included some interesting data on toxicity, especially in relation to cisPDD. Ultrastructural changes due to Mitomycin C are outlined by Y. Daskal and S. T. Crooke, but the quality of the plates of transmission electron microscopy is not quite good enough to discern the nucleolar segregation alluded to. A short and interesting article on suppressor activity in the spleen of mice, by B. F. Argyris, seems curiously irrelevant to a book on Mitomycin C, as the drug is not mentioned. The potentiation of Mitomycin C activity by the DNA-repair inhibitor, leucanthone, is described by L. S. Koons and co-workers.

The remainder of the book gives detailed summaries of the various clinical aspects of the drug. The first on Acute Intermittent Dose Scheduling (L. H. Baker and V. K. Vaitkevicius) points out the advantage of giving a series of short sharp shocks rather than a single or continuous treatment. The succeeding chapters deal separately with the single agent and different combinations of agents, including Mitomycin and Adriamycin (R. L. Comis *et al.*) especially in advanced breast cancer (L. R. Morgan) where carbotoxicity proves to be a problem. This could be compared with a chapter on Mitomycin C alone (T. E. Godfrey). Mitomycin alone and in combination with Adriamycin and cyclophosphamide are compared in advanced adenocarcinoma of the lung (M. K. Samson *et al.*) as a single agent in gastric cancer (R. L. Comis), combinations of 5FU, Adriamycin and Mitomycin C are compared with Streptomycin, Mitomycin C and 5FU, and of these the first combination (FAM) proved to be the most useful in the Phase II trials discussed (P. S. Schein *et al.*). Other chapters include combination studies in gastric carcinoma (P. J. Panettiere & L. Heilbrun), a single-drug study with cervical cancer in the U.S. (L. H. Baker), metastatic cervical cancer with sequential Bleomycin and Mitomycin C (T. Miyamoto), single agent in Head and Neck cancer in Japan (Y. Inuyama), a combination of Mitomycin C, 5FU and X-radiation in squamous (epidermoid) cell carcinoma of the anal canal, pre-operatively (T. Buroker *et al.*), single agent in epidermoid cancer of the lung (L. S. Koons *et al.*), Mitomycin C instillation in bladder tumours (T. Mishina & H. Watanabe) and multiple superficial tumours of urinary bladder with intravesical Mitomycin C (R. B. Braken & D. Johnson), M. L. Gutierrez reviews the use of Mitomycin C in childhood cancer, and V. Ratanatharathorn *et al.* reviews the nephrotoxic activity of Mitomycin C in patients. Pulmonary toxicity (S. Mortino *et al.*) and clinical pharmacology (S. D. Reich) are discussed before the final summary chapter by S. K. Carter, in which he concludes that the agent is active in solid tumours, with a broad spectrum, and offers exciting potentials for combination study. This book is a significant addition to the library of both experimental and medical oncologists.

B. W. Fox